# Teaching next-generation sequencing to medical students with a portable sequencing device

**DOI:** 10.1007/s40037-020-00568-2

**Published:** 2020-03-03

**Authors:** Jorge Cervantes, Cynthia Perry, Min Chih Wang

**Affiliations:** Paul L. Foster School of Medicine, Texas Tech University Health Sciences Center, El Paso, USA

**Keywords:** Next generation sequencing, Medical education, Training, New technologies

## Abstract

**Background:**

There continues to be a disjoint between the emergence of new diagnostic technologies and venues to train new physicians on how to apply them. Next-generation sequencing (NGS) has become a very important tool for a wide range of clinical applications. Technical complexity and cost have been the major obstacles in incorporating these technologies into the classroom.

**Goal for innovation:**

We opted to use the MinION, which is a new portable DNA sequencer that can produce data in real-time at a relatively low cost, for a NGS hands-on workshop with medical students.

**Steps taken:**

We conducted a pilot NGS hands-on practical module in order to expose an interested group of medical students to this new portable sequencer device. A pre- and post-survey, using a Likert-type scale survey items and open-ended questions, evaluated participant resistance to new diagnostic tools, familiarity with NGS, and likelihood to use a portable sequencer in clinical practice.

**Outcomes:**

Prior to participating in our learning workshop, students did not understand how to incorporate NGS into clinical practice, and expressed that cost and prior training/knowledge were among the limiting factors in their likelihood to use NGS as a diagnostic tool. After participating in the module, students’ responses demonstrated a shift in their understanding of the scientific principles and applications of NGS (pre- and post-survey scores *p* < 0.05).

**Reflection:**

The hands-on experience not only helped students become closer to and more comfortable with NGS, but also served as a venue to discuss the science and application of this technology in medicine. Such discussion helped to provide participants with current “genetic literacy” that is often incompletely covered in the typical undergraduate medical education curriculum.

**Electronic supplementary material:**

The online version of this article (10.1007/s40037-020-00568-2) contains supplementary material, which is available to authorized users.

## Background and need for innovation

Historically, advanced diagnostic tools have been accessible only through clinical diagnostic testing labs, while physicians, medical students, and resident trainees would have little to no access to or understanding of their use. These tools are becoming more common and their costs are beginning to drop below current gold standards, further reducing the barrier to clinical application. Despite this, there continues to be a disjoint between the emergence of new diagnostic technologies and venues to train new physicians on how to apply them [1].

Next-generation sequencing (NGS) has become the most important tool for a wide range of clinical applications, including bacterial identification, antibiotic resistance detection, non-invasive prenatal testing, tumor profiling, and genetic screening [2–4]. NGS technologies have transformed sequencing from a method practiced only by experts in dedicated facilities to a general purpose research tool available to researchers in a wide range of fields [5]. The growing gap between the demand for genome sequencing and the supply of trained genomics professionals is creating an acute need to develop more effective genomics education [6].

As a first step toward developing a robust curriculum that includes new technological advances, our institution has implemented several 2‑hour sessions given to 1st and 3rd year medical students, covering themes such as “Therapeutic use of stem cells”, “Cancer immunotherapy”, and “CRISPR technology”. Although NGS is mentioned in some of these sessions, there is currently no opportunity to dive into the applications of this technology. A practical approach, consisting of a quick and simple protocol with real-time visualization of the ongoing sequencing process, has the advantage of creating a bridge to overcome fear of a technology that may be seen to be too complex or esoteric.

## Goal of innovation

Given the ongoing and rapid advancements in diagnostic technologies, it is our duty as medical educators to prepare medical students to identify and be able to use these tools. The incorporation of these topics into the classroom has, however, been challenging, due to an implied high degree of technical complexity [7], and costs. Previous initiatives to bring NGS technology to medical school [6] and high school students [8], although successful in promoting scientific research, require funding due to the costs of the sequencing platforms used [5, 8]. In fact, the greatest impasse for educational use of NGS remains the cost, at approximately $4000 per experiment using an Illumina MiSeq (excluding the capital expense of equipment, approximately $100,000) [5]. A low-cost approach proposed the use of existing materials created by biotechnology companies to compare their instrumentation and chemistries [7]. Virtual attempts to teach NGS used online simulation modules [9]. These approaches, however, lacked the hands-on variable, affecting experiential learning.

The MinION (Oxford Nanopore Technologies) is a new low capital cost portable DNA sequencer, approximately the size of a flash drive, which can produce data in real-time [10] (see the Appendix in the Electronic Supplementary Material). The emergence of novel portable sequencers promises to decrease costs for reagents and instrumentation [11]. This device provides high-accuracy sequence data, with various types of samples [12, 13]. The company also provides a set of applications for rapid and accurate bacterial identification [14]. For all this, we decided to use this low-cost, easy-to-use device as a means of delivering a short and practical module to expose students to NGS via experiential learning.

## Steps taken for development and implementation of innovation

The MinION seemed to be an ideal option to reach our goal of a low-cost platform to be used for a hands-on experience on NGS with our students. With this in mind, we conducted our first NGS hands-on practical module at the Laboratory for Education in Molecular Medicine, a dual function teaching and research lab, in order to expose an interested group of medical students to a new portable sequencer device. The manufacturing company (Oxford Nanopore) supports educational initiatives using the MinION, and kindly lent us extra sequencers and reagents for our workshop. Due to the gap in knowledge in regards to both NGS and potential downstream applications, Oxford Nanopore wants more learners to be exposed to DNA sequencing through hand-on opportunities and trainings.

We set up a NGS hands-on practical module using the MinION to provide a group of medical students with a first-hand experience of the latest DNA sequencing technology, while learning about the principles of DNA sequencing and DNA extraction [15]. The activity was voluntary, and consisted of three main sections: A brief review of DNA and sequencing concepts, followed by a practical hands-on experience on library preparation and flow-cell loading (see the Appendix in the Electronic Supplementary Material), and ended up with a discussion on the application of NGS in medicine, once the sequencing started to run.

## Outcomes of innovation

A previous report of an activity letting medical students sequence their own genome at no cost showed that they were more persistent in overcoming the practical challenges of genome analysis and had a better understanding of the patient experience as a result of working with their own genome [6]. The study, however, could not quantify whether analyzing your own genome improved educational outcomes [6].

We evaluated the impact of our practical module on participants’ understanding of NGS and its applicability in medicine; we developed a survey in order to compare participants’ perceived preparedness and receptiveness towards NGS before and after the activity. The survey was administered before and after the activity and contained 10 Likert-type scale survey items and 2 open-ended questions, evaluating participant resistance to new diagnostic tools, familiarity with NGS, and likelihood to use NGS in clinical practice (Table 1). The Likert-type scale items had 4 response levels either requiring respondents to qualify their level of satisfaction on a certain topic while others asked about level of agreement with a certain concept.

**Tab. 1:** Box Survey questions

1.	I understand the scientific principles behind NGS
2.	I understand how I can incorporate NGS into my clinical duties
3.	I am likely to use new diagnostic technology like NGS in my practice
4.	NGS is a practical tool for in office clinical diagnostic use
5.	There is a gap in training provided to physicians regarding emerging diagnostic technology
6.	I feel comfortable selecting the most appropriate common diagnostic and screening tests
7.	I feel comfortable interpreting common diagnostic and screening tests
8.	I feel comfortable selecting the most cost-effective diagnostic and screening tests
9.	I feel that diagnostic testing should be done in a lab and not in the clinic
10.	Having a better understanding of emerging diagnostic technologies such as NGS will improve my academic and clinical practices

The items were selected from previously published surveys or designed and approved after peer review and examination for scope and clarity of purpose. An Institutional Review Board exemption was obtained. Responses to the Likert-type scale items were converted from ordinal to numerical scale for the purposes of statistical analysis [16]. Open-ended survey items could not be grouped for analysis. To evaluate the responses as a whole, total pre- vs. post-survey score were compared, utilizing t‑test, as data passed the Shapiro-Wilk and Kolmogorov-Smirnov normality tests. Each item of the survey converted numerically was also subjected to comparison in the same manner.

**Fig. 1:**
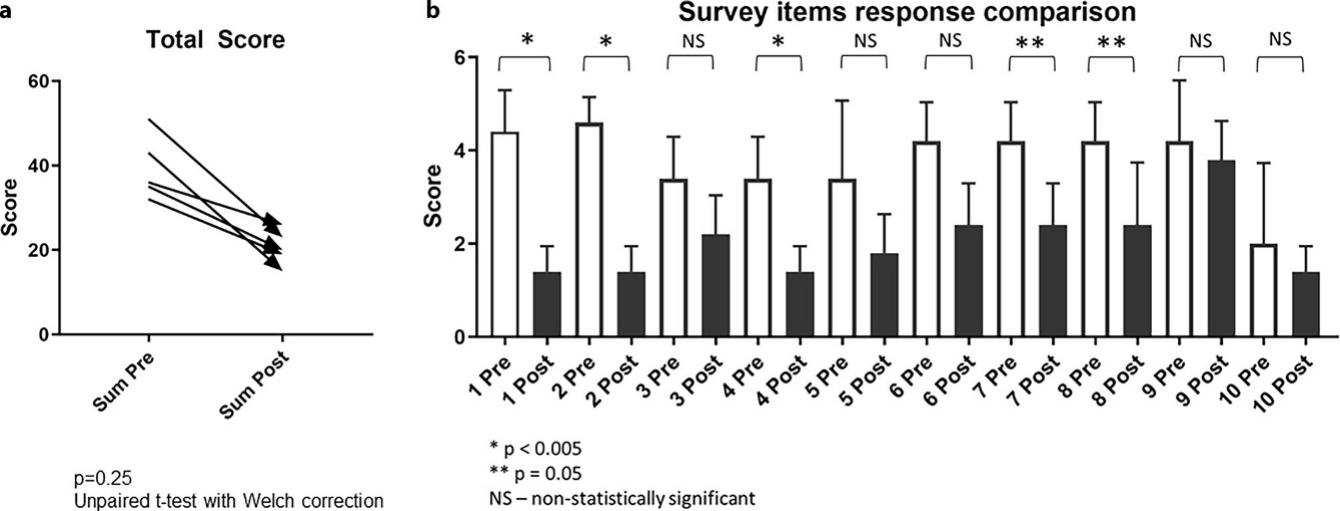
**a** Pre- vs. post-survey scores **b** Survey item comparison (pre- vs. post-workshop)

Prior to participating in our learning workshop, students did not understand how to incorporate NGS into clinical practice, and expressed that cost and prior training/knowledge were among the limiting factors in their likelihood to use NGS as a diagnostic tool. After participating in the module, students’ responses demonstrated a shift in their understanding of the scientific principles and applications of NGS (pre- and post-survey scores *p* < 0.05) (Fig. 1a). Areas where a significant difference was observed included understanding the scientific principles behind NGS, how to incorporate NGS into clinical duties, how to use it as a clinical diagnostic tool, interpretation of common diagnostic and screening tests, and selecting the most cost-effective diagnostic and screening tests (Fig. 1b).

## Critical reflection on your process

Even though validity testing was not performed on the utilized tool, we developed it using several items taken from previously published and validated questionnaires, as well as new items that were “validated” through peer review to determine if they were clearly worded and addressed what we were trying to enquire about. The outcome reflects that the initiative was indeed useful. Our workshop helped students to understand how to incorporate NGS into clinical practice. Feedback such as “I now understand how I can incorporate NGS into my clinical duties” and “I have a better understanding of the scientific principles behind NGS”, were found among the open-ended questions in the survey. Not only did the hands-on experience help students become closer and more comfortable with NGS, it also served as a venue to discuss the science and application of this technology in medicine. Such discussion helped to provide participants with current “genetic literacy” that is often incompletely covered in the typical undergraduate medical education curriculum.

Still, the obstacle of easier data analysis needs to be overcome, as a certain level of computational skills are needed to process NGS data.

Creation of innovative educational opportunities for medical students and residents to increase their exposure and understanding of state of the art sequencing technologies constitutes a feasible proposal with great potential to face gaps in medical education.

## Caption Electronic Supplementary Material

**Appendix. A.** Portable sequencing device (MinION, Nanopore Technologies). **B** MS1 demonstrating flow-cell loading of the DNA to be sequenced. During the workshop students sequenced genomic DNA from different strains of *Mycobacterium tuberculosis*
