# Supplementary figures and images for: Teaching next-generation sequencing to medical students with a portable sequencing device
Source: Perspect Med Educ. 2020 Mar 3;10(4):252–5. doi: 10.1007/s40037-020-00568-2 (PMC8368599; doi:10.1007/s40037-020-00568-2)

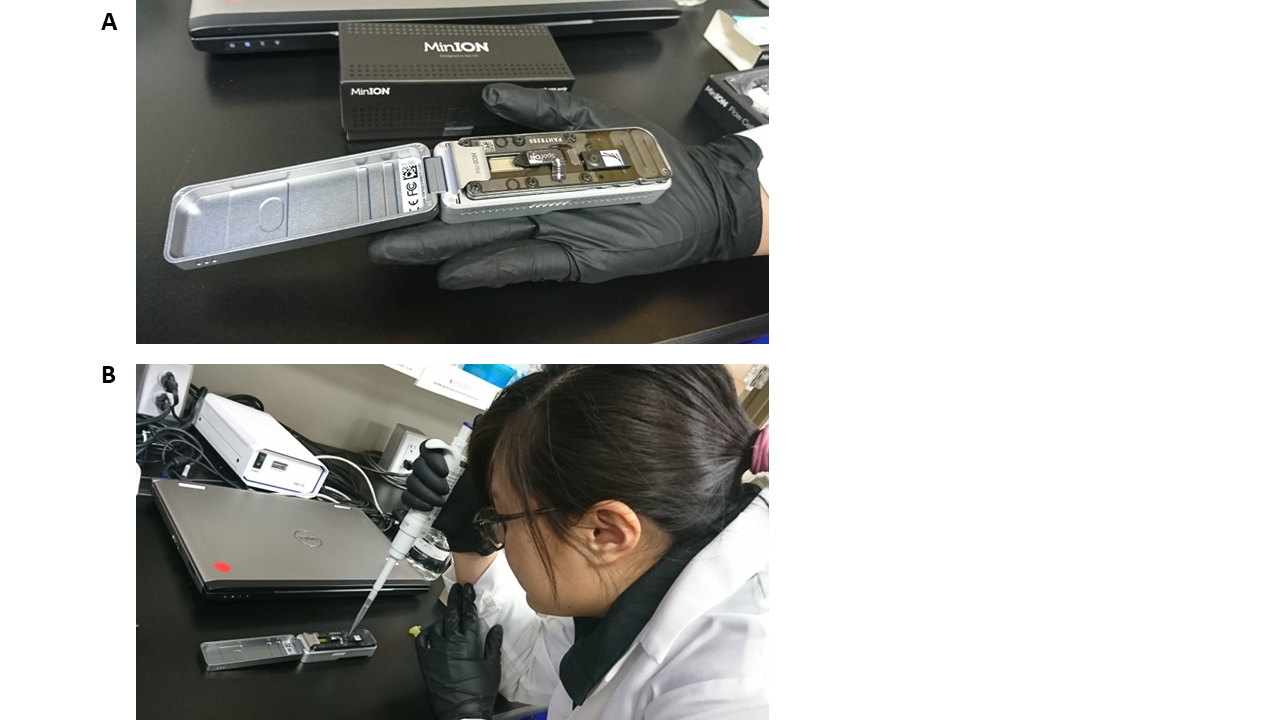

Supplement: Supplementary file 1 — Appendix. A. Portable sequencing device (MinION, Nanopore Technologies). B MS1 demonstrating flow-cell loading of the DNA to be sequenced. During the workshop students sequenced genomic DNA from different strains of Mycobacterium tuberculosis [file 40037_2020_568_MOESM1_ESM.tif]
